# A Single‐Molecule Pleuripotent Scaffold for Combinational Therapy of Alzheimer's Disease via Intranasal Administration

**DOI:** 10.1002/advs.202511611

**Published:** 2025-08-27

**Authors:** Jian‐Mei Gao, Wen‐Bo Li, Na‐Na Chen, Yang Yi, Ze‐Han Wang, Xian Chen, Yang‐Yang Zhao, Ze‐Li Yuan, Jie Gao, Yu‐Chen Pan, Dong‐Sheng Guo, Qi‐Hai Gong

**Affiliations:** ^1^ School of Pharmacy Key Laboratory of Basic Pharmacology of Ministry of Education and Joint International Research Laboratory of Ethnomedicine of Ministry of Education Zunyi Medical University Zunyi 563000 China; ^2^ College of Chemistry State Key Laboratory of Elemento‐Organic Chemistry Key Laboratory of Functional Polymer Materials (Ministry of Education) Frontiers Science Center for New Organic Matter Collaborative Innovation Center of Chemical Science and Engineering (Tianjin) Nankai University Tianjin 300071 China; ^3^ Key Laboratory of Macrocyclic and Supramolecular Chemistry of Guizhou Province Guizhou University Guiyang 550025 China; ^4^ National Engineering Research Center of Pesticide Nankai University Tianjin 300071 China

**Keywords:** Alzheimer's disease, combinational therapy, intranasal administration, macrocyclic hosts, supramolecular materials

## Abstract

The intricate pathological mechanisms of Alzheimer's disease (AD), along with the restrictive nature of the blood‐brain barrier (BBB) that further impedes the drug brain entry, underscore the pressing need for innovative combinational therapy to achieve effective treatment outcomes. Intranasal administration, capable of bypassing BBB by direct transport through olfactory and trigeminal nerves, provides a promising approach for treating neurological disorders. Herein, the guanidinium‐modified calix[5]arene (GC5AY) is developed as a single‐molecule pleuripotent scaffold, demonstrating small size, positive charge and desirable amphiphilicity, which facilitate its efficient traverse of nasal mucosal barrier. The multifunctionality of GC5AY, including inhibiting amyloid fibrosis, scavenging reactive oxygen species and drug delivery, enables it to serve as a sophisticated platform for constructing multi‐target AD therapeutic agents. In light of this, by loading neuroprotective agent Trilobatin (TLB) into the cavity of GC5AY, intranasal administration of the TLB@GC5AY formulation is verified to effectively attenuate the cognitive impairment of AD mice, demonstrating multifaceted pathological improvements, while also possessing good biocompatibility. In response to the growing appeal for combinational therapy of AD, the approach proposed in this study has provided a readily generalizable strategy to fulfill this pursuit.

## Graphical abstract

Herein, guanidinium‐modified calix[5]arene (GC5AY) is developed featuring small size, positive charge, and desirable amphiphilicity, that can efficiently traverse the nasal mucosal barrier for brain drug delivery. Additionally, possessing inherent therapeutic functions, GC5AY can thus serve as a single‐molecule pleuripotent scaffold for multi‐target combinational therapy of Alzheimer's disease via intranasal administration.

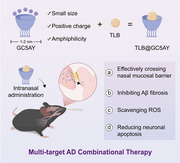

## 1 Introduction

Alzheimer's disease (AD) represents a complex neurodegenerative condition pathologically defined by three cardinal manifestations, including extracellular deposition of β‐amyloid peptides forming senile plaques, intracellular accumulation of hyperphosphorylated tau proteins constituting neurofibrillary tangles, and progressive neuronal loss.^[^ ^1^ ^]^ Contemporary clinical management of AD continues to rely predominantly on cholinesterase inhibitors and N‐methyl‐d‐aspartate receptor antagonist, which were developed decades ago and primarily focused on symptomatic management.^[^ ^2^ ^]^ The United States Food and Drug Administration has authorized three anti‐amyloid monoclonal antibodies (i.e., Aducanumab, Lecanemab, and Donanemab) through accelerated approval pathways since 2021, establishing the first pharmacological class with potential disease‐modifying effects in AD therapeutics.^[^ ^3^ ^]^ Notably, AD demonstrates a multifactorial pathogenesis,^[^ ^4^ ^]^ characterized by multiple interconnected pathological processes.^[^ ^4^, ^5^ ^]^ The neurodegenerative progression is driven by amyloidogenic β‐amyloid (Aβ) fibrillization synergized with transition metal dysmetabolism oxidative stress through increasing reactive oxygen species (ROS),^[^ ^6^ ^]^ neuroinflammation, tau hyperphosphorylation, cholinergic dysfunction, progressive synaptic deterioration and so on.^[^ ^6^, ^7^ ^]^ Recognition of this complexity suggests that, simultaneously addressing more than one target in the form of combinational therapy is critically necessary for AD treatment,^[^ ^8^ ^]^ which has emerged as a developmental trend in nowadays investigations.

The blood‐brain barrier (BBB) exhibits another formidable pharmacological obstacle in AD's therapeutics, with its sophisticated physical, transport, and metabolic disturbance limiting the effect of peripherally administered pharmaceuticals such as intravenous and oral administration.^[^ ^9^ ^]^ Intranasal administration constitutes a translational breakthrough in central nervous system (CNS) therapeutics offering obvious advantages including non‐parenteral transmucosal delivery,^[^ ^10^ ^]^ bypass of first‐pass effect, and fast‐acting therapeutic onset.^[^ ^10^, ^11^ ^]^ Notably, this approach promotes direct drug transport to the CNS through the olfactory and trigeminal nerves connecting the brain and the nasal cavity, presenting an effective approach for bypassing BBB.^[^ ^12^ ^]^ Pharmacokinetic studies demonstrate that, according to the physicochemical properties of the drug, the brain absorption efficiency of intranasal administration exhibits several to hundreds of folds superior compared to systemic intravenous administration.^[^ ^13^ ^]^ Currently, intranasal insulin is utilized to treat AD in clinical trials, indicating the significant potential of intranasal administration.^[^ ^14^ ^]^ The therapeutic effect of intranasal administration depends on several factors: 1) the nasal mucosal epithelial cells establishes a size‐selective paracellular barrier through tight junctions, effectively impeding passive diffusion of macromolecular therapeutics and nanoparticulate systems;^[^ ^15^ ^]^ 2) cationic nanocarriers can interact with the anionic phospholipid bilayer through electrostatic interaction, thereby enhancing their adhesion to the mucosal surface and improving the transmembrane efficiency;^[^ ^16^ ^]^ 3) appropriate amphiphilicity enables the formulation to effectively penetrate the lipid bilayer of nasal epithelial cells via passive membrane diffusion, promoting drug permeation across the mucosa.^[^ ^17^ ^]^ Collectively, a formulation integrated with small size, positive charge, and amphiphilicity is highly desired for the intranasal administration.^[^ ^18^ ^]^

**Scheme 1:**
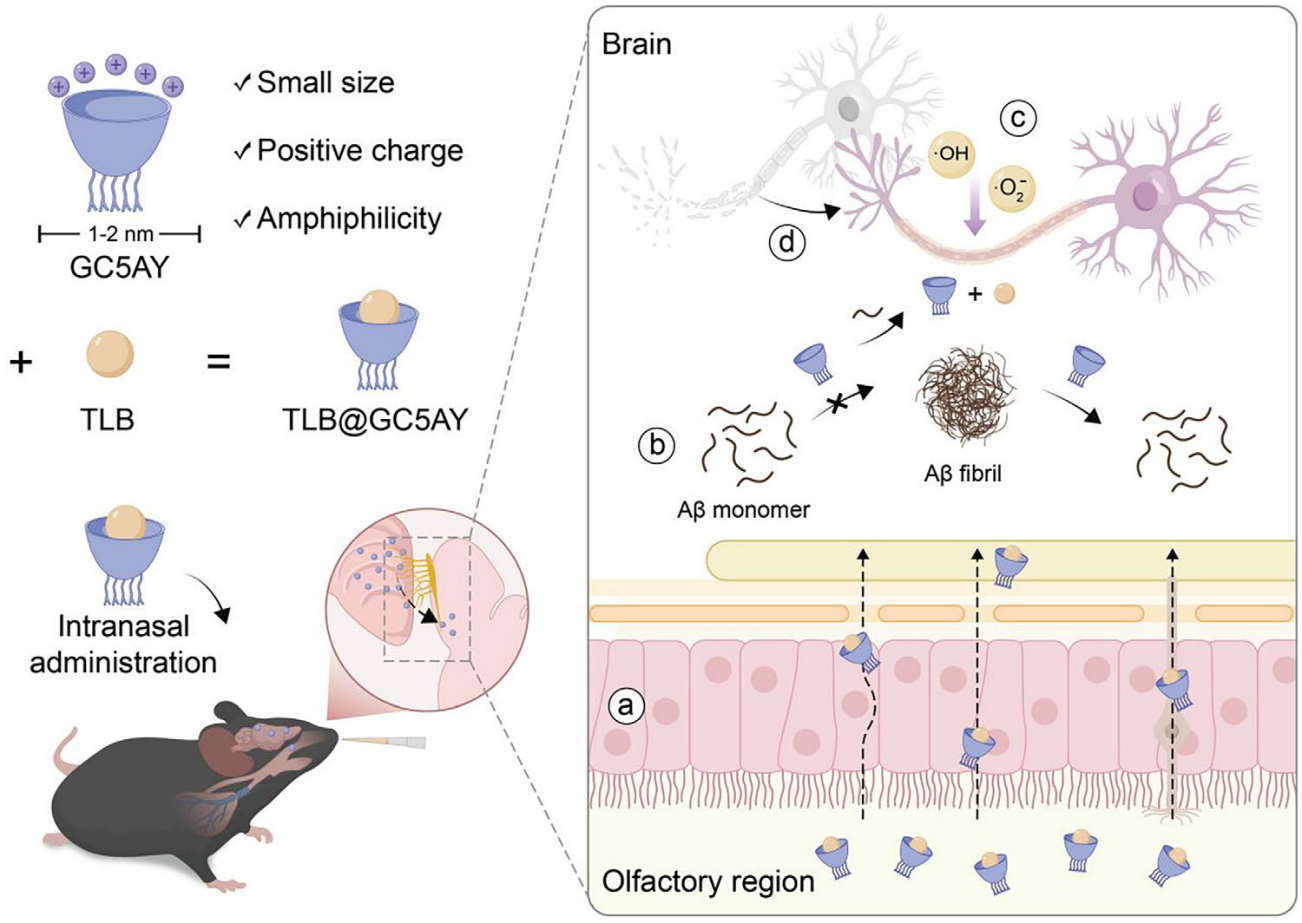
Illustration of GC5AY as a single‐molecule pleuripotent scaffold for combinational therapy of AD via intranasal administration. TLB@GC5AY was capable of effectively a) traversing the nasal mucosal barrier, b) inhibiting Aβ aggregation and disintegrating the mature fibrils, c) reducing ROS and oxidative stress, as well as d) suppressing neuronal apoptosis.

In light of these, we herein developed a guanidinium‐modified calix[5]arene (GC5AY) as a single‐molecule operating scaffold for intranasal administration in AD combinational therapy (**Scheme** **1**). Primarily, GC5AY with five upper‐rim guanidinium groups and five lower‐rim alkyl chains exhibited small size in dimension (1 − 2 nm), positive charge and desirable amphiphilicity,^[^ ^19^ ^]^ which collectively facilitated its easy traverse of the nasal mucosal barrier. As considering the inherent encapsulation property of macrocyclic host,^[^ ^20^ ^]^ GC5AY could therefore serve as a single‐molecule carrier for efficiently delivering drugs through intranasal administration. Additionally, GC5AY also demonstrated multifaceted therapeutic functions for AD. Specifically, 1) the high binding affinity of GC5AY toward Aβ enabled it to effectively inhibit Aβ aggregation and promote the disintegration of mature fibrils; 2) the skeleton of calixarene endowed GC5AY with inherent ROS scavenging capability,^[^ ^21^ ^]^ which contributed in alleviating oxidative stress and neuroinflammation within the AD brain. Leveraging these attributes, we chose trilobatin (TLB), a recognized neuroprotective agent capable of anti‐Aβ deposition, anti‐oxidation, and anti‐inflammation,^[^ ^22^ ^]^ as the model drug loaded in the cavity of GC5AY to prepare the TLB@GC5AY formulation. Given the integration of efficient intranasal delivery mediated by the structural features of GC5AY and the advantages of multi‐targeted combinational therapy, TLB@GC5AY has been confirmed to obviously attenuate the cognitive impairment in AD mice, with marked reduction of Aβ burden, oxidative stress, neuroinflammation, mitochondrial damage, and neuronal apoptosis. This study offers a novel supramolecular strategy for designing drug carriers for intranasal delivery and multi‐targeted combinational therapy of AD, which is of great potential to promote the treatment development for such complex neurological disorders.

## 2 Results and Discussion

### 2.1 Structure and Recognition Characterizations of GC5AY

**Figure 1:**
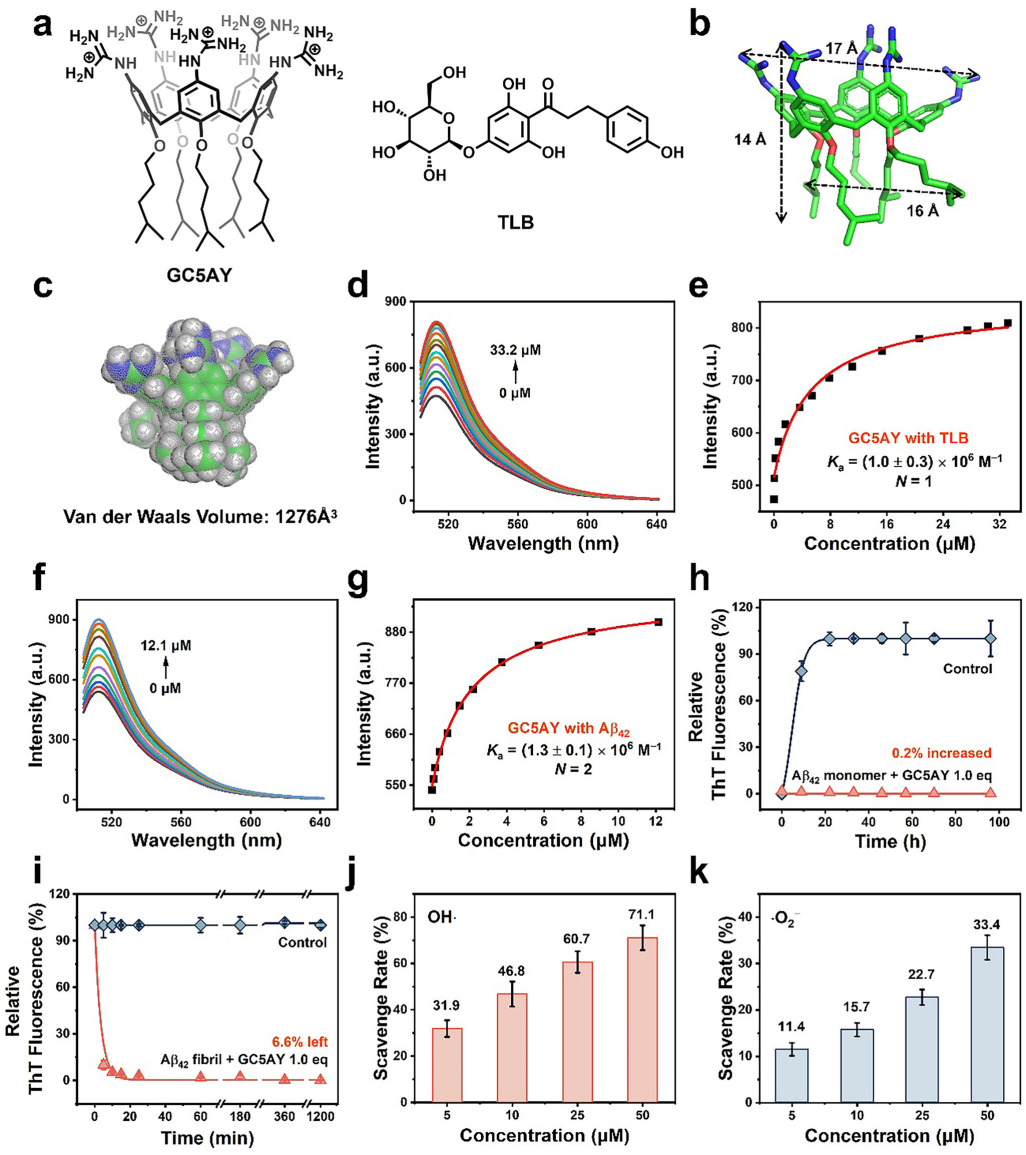
Characterizations on the structure, recognition properties, and therapeutic functions of GC5AY. a) The chemical structures of GC5AY and TLB. b) The geometry optimized structure of GC5AY at the B3LYP/6‐31G(d) level, and c) the occupied volume of GC5AY calculated by Molo Vol. Competitive fluorescence titration of d) TLB and f) Aβ_42_ in the presence of Fl and GC5AY, and the corresponding titration curve for e) TLB and g) Aβ_42_, fitted according to the *N*:1 binding stoichiometry. Note: The *N* for GC5AY and Aβ_42_ was fixed as 2, which means that there were two GC5AY together bond with one Aβ_42_. And since the *K*_a_ for one GC5AY binding with one Aβ_42_ was (1.3 ± 0.1) × 10^6^ M^−1^, the overall *K*_a_ between GC5AY and Aβ_42_ was obtained by squaring this value, and determined to be (1.7 ± 0.3) × 10^12^ M^−2^.^[^ ^26^ ^]^ The relative fluorescence of ThT of h) Aβ_42_ monomer, and i) pre‐formed Aβ_42_ fibrils co‐incubated with or without GC5AY. The normalized scavenge rate of j)·OH and k) O_2_^−^ for GC5AY. The data were expressed as mean ± s.d. from three independent experiments.

GC5AY was synthesized according to the previously reported procedures.^[^ ^19a^ ^]^ First, the logarithmic value of oil‐water partition coefficient (logP) for GC5AY was evaluated to be 0.48 ± 0.06 (Figure S1, Supporting Information), indicating the anticipated amphiphilicity that was desirable in traversing the nasal mucosal barrier. (Generally, molecules with logP value ranging 0 − 5 exhibit better permeability to cell membrane. Such a moderate LogP value simultaneously meet the requirements for 1) appropriate lipophilicity (logP > 0) to facilitate partitioning into epithelial membranes, and 2) sufficient aqueous solubility (logP < 5) to ensure dissolution in the mucus layer.^[^ ^23^ ^]^). And the critical aggregation concentration (CAC) of GC5AY was reported to be ≈400 µm.^[^ ^24^ ^]^ This value was much higher than the concentration used in the following experiments, implying the single‐molecule state of GC5AY under the conditions. Furthermore, as referred to the geometry optimized structure, GC5AY was measured to be 16.6 Å in length, 15.7 Å in width, 14.4 Å in height with a Van der Waals volume of 1276 Å^3^ (**Figure** **1**b,c). And the diffusion coefficient (*D*) of GC5AY with a concentration of 100 µm below CAC, was determined to be 1.76 × 10^−10^ m^2^ s^−1^ according to 2D diffusion‐ordered nuclear magnetic resonance spectroscopy measurement (Table S1, Supporting Information). Its molecular diameter was then calculated to be 13.9 Å based on the Einstein's diffusion law, which was consistent with the value obtained from geometry optimized structure.^[^ ^25^ ^]^ Such a small size dimension facilitated GC5AY in traversing the nasal mucosal barrier as a single‐molecule carrier.

Subsequently, the binding affinities of GC5AY for both TLB and Aβ_42_ were measured through competitive fluorescence titrations using fluorescein (Fl) as a reporter dye (Figure S2, Supporting Information), and further fitted out according to the *N*:1 binding stoichiometry.^[^ ^26^ ^]^ As illustrated in Figure 1d,e, the binding constant (*K*_a_) between GC5AY and TLB was determined to be (1.0 ± 0.3) × 10^6^ M^−1^ (*N* = 1), confirming the anticipated encapsulation. And for Aβ_42_, the value was (1.7 ± 0.3) × 10^12^ M^−2^ (*N* = 2, refer to Figure 1g legend for detail notes), which suggested that GC5AY could effectively bind to Aβ_42_ (Figure 1f,g). Therefore, in virtue of the biomarker displacement activation strategy,^[^ ^24^ ^]^ TLB could be responsively released when meeting the overproduced Aβ_42_ in AD brain through competing the host cavities with them, thus achieving precise drug delivery.

### 2.2 Inhibiting Fibrillation and Scavenging ROS of GC5AY

As given the verified binding of GC5AY with Aβ_42_, the capability of GC5AY on inhibiting Aβ_42_ fibrillation and disintegrating the mature fibrils was investigated next, first by thioflavine T (ThT) assay, which is a fluorescent probe primarily used in monitoring the content of β‐sheet in proteins or peptides.^[^ ^27^ ^]^ As showed in Figure 1h, significant increase of ThT fluorescence was observed in the control group after 96 h, indicating the formation of abundant Aβ_42_ fibrils rich in β‐sheet structure. While almost no increase (only 0.2%) was noticed upon introduction of equimolar quantity of GC5AY to Aβ_42_, demonstrating its complete inhibition on the Aβ_42_ fibrillation. More thrillingly, the pre‐formed Aβ_42_ fibrils could be rapidly disintegrated by GC5AY, as evidenced by a near 90% reduction in ThT fluorescence within mere 5 min co‐incubation (Figure 1i). And this process of disintegration was virtually complete, with only 6.6% fluorescence persisting at the end of test. Subsequently, the morphological alterations of Aβ_42_ through co‐incubation were characterized using transmission electron microscope (TEM) (Figure S3, Supporting Information). In the control group without GC5AY added, abundant fibrils could be clearly detected after 96 h. While the addition of GC5AY (equal equivalent of Aβ_42_) significantly inhibited the fibril generation, with nearly no visible fibril in the visual field. While in the disintegration process, GC5AY under the same concentration was observed inducing complete disintegration of the pre‐formed fibrils. These findings were consistent with ThT assay results, jointly validated the excellent efficiency of GC5AY in both inhibition of Aβ_42_ aggregation and disintegration of mature fibrils.

Additionally, as considering the ROS scavenging capability of calixarene skeleton previously reported,^[^ ^21a^ ^]^ the antioxidant efficiency of GC5AY was further examined. Two typical ROS was selected for assessment, the highly reactive hydroxyl radical (OH⋅), and the biologically abundant superoxide anion (⋅O_2_^−^). The scavenging efficiency was quantitatively measured using established chemical assays, where OH⋅ was produced through the H_2_O_2_/ Fe^2+^ Fenton reaction, and ⋅O_2_^−^ was generated via the xanthine oxidase mediated catalysis of xanthine (see supporting information for detailed methodology). As illustrated in Figure 1j,k, pronounced elimination effect was observed for GC5AY, with a substantial reduction of 71.1% for OH⋅ and 33.4% for ⋅O_2_^−^ upon the addition of 50 µm. This was consistent with the robust ROS scavenging efficiency of GC5A12C (possessing same guanidinium modification with GC5AY on the upper rim, while twelve carbon atoms per alkyl chain) (Figure S4a, Supporting Information) observed in our previous study,^[^ ^28^ ^]^ where it demonstrated a 78.6% reduction for OH⋅ and 42.8% for ⋅O_2_^−^, respectively, under the same concentration, even surpassing the efficiency of ascorbyl palmitate (a l‐ascorbyl‐based antioxidant compound with pronounced reducing nature), as 60.9% for OH⋅ and 12.0% for ⋅O_2_^−^, respectively. This excellent antioxidant potential of GC5AY established a foundation for ameliorating the oxidative stress in AD.

### 2.3 Traversing the Nasal Mucosal Barrier of GC5AY

**Figure 2:**
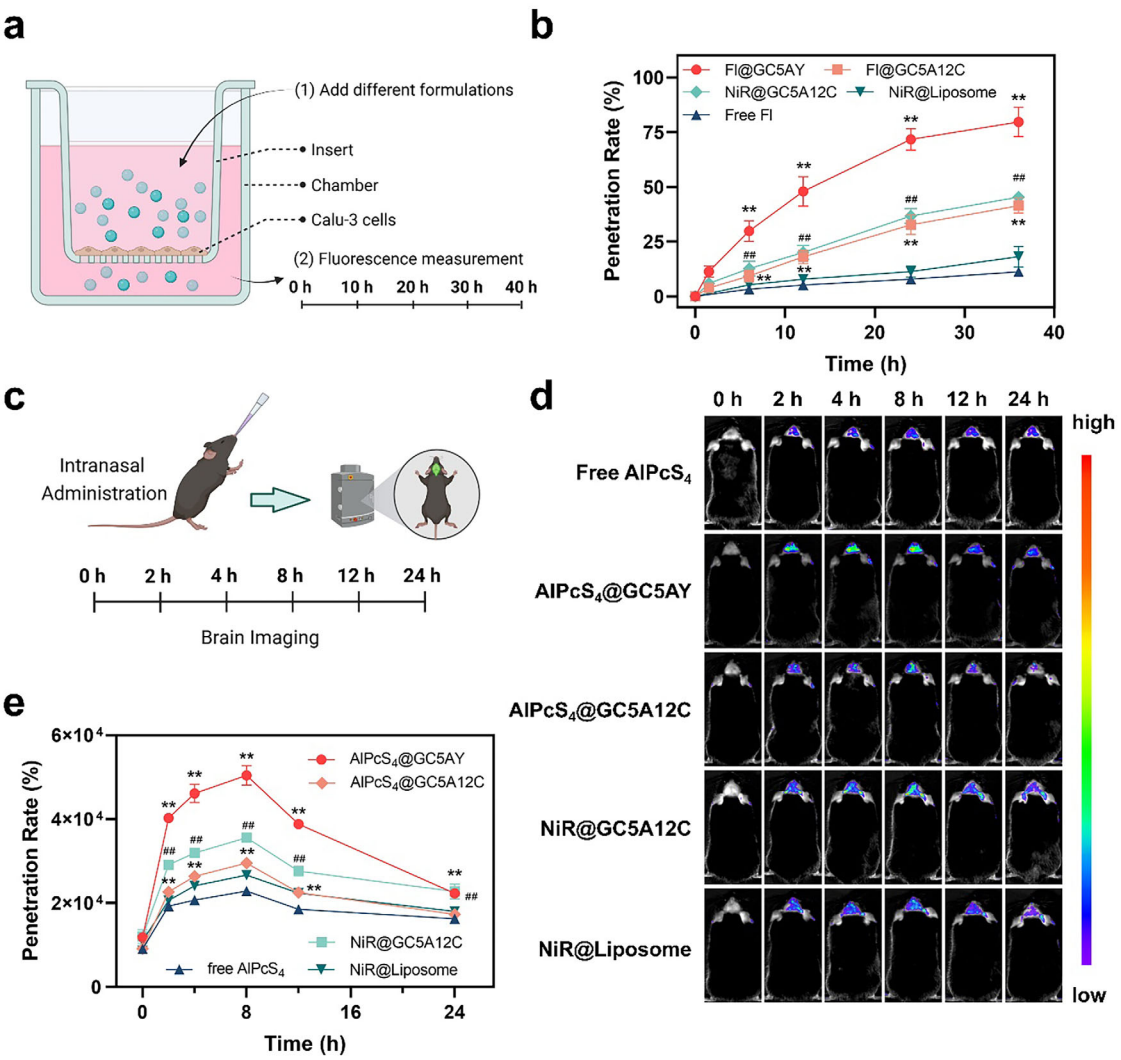
The brain delivery efficiency of GC5AY via intranasal administration. a) The illustration of transwell experiment. b) Penetration rate of different carriers in the transwell experiment. The data were expressed as mean ± s.d. from three independent experiments. c) Time schedule of in vivo imaging protocol. d) Representative fluorescent images of different formulations in in vivo imaging. e) Quantification of the average fluorescent intensity of different formulations in in vivo imaging. The data were expressed as mean ± s.d. from five mice in one group. The significance levels, with ^**^ *p* < 0.01 versus free‐AlPcS_4_ and ^##^ *p* < 0.05 versus NiR@Liposome, were analyzed by one‐way ANOVA with Tukey's test.

To evaluate the efficiency of GC5AY for traversing the nasal mucosal barrier, we initially performed the transwell experiments, utilizing Calu‐3 cells for establishing an in vitro model.^[^ ^30^ ^]^ First, GC5A12C was employed as an assembly carrier for comparison, which demonstrated a diameter of 42.9 nm (Figure S4b, Supporting Information). As shown in **Figure** **2**a,b, through loading Fl into the cavities of hosts as an indicator, GC5AY was observed exhibiting a nearly two‐fold higher penetration rate than GC5A12C at 36 h. Such a result could be attributed to the smaller size of GC5AY, which facilitated traversing the barrier. Furthermore, the penetration rates of GC5A12C and liposomes prepared by 1,2‐distearoyl‐sn‐glycero‐3‐phosphorylcholine were compared through loading nile red (NiR) in the intermolecular space, where GC5A12C with positive charges demonstrated a 2.5‐fold better efficiency than liposomes. Hence, it could be concluded that GC5AY exhibited the highest penetration rate, surpassing both GC5A12C and liposomes.

Subsequently, we further performed in vivo fluorescence imaging for examining the brain delivery efficiency of these formulations via intranasal administration (Fl was replaced with AlPcS_4_, as dye with long emission wavelength was needed for in vivo imaging).^[^ ^31^ ^]^ As illustrated in Figure 2c−e, both GC5AY and GC5A12C facilitated the entry of AlPcS_4_ into the brain, with the fluorescence intensity in these two groups significantly higher than free AlPcS_4_ within 24 h. While GC5AY surpassed GC5A12C, and similarly, GC5A12C showed a better performance than liposomes, which was consistent with the result in in vitro experiment. Therefore, as a single‐molecule carrier, GC5AY possessed obvious advantages in traversing the nasal mucosal barrier, which facilitated the effective brain drug delivery via intranasal administration.

### 2.4 Ameliorating Cognitive Impairment in 3×Tg AD Mice via Intranasal Administration of TLB@GC5AY

**Figure 3:**
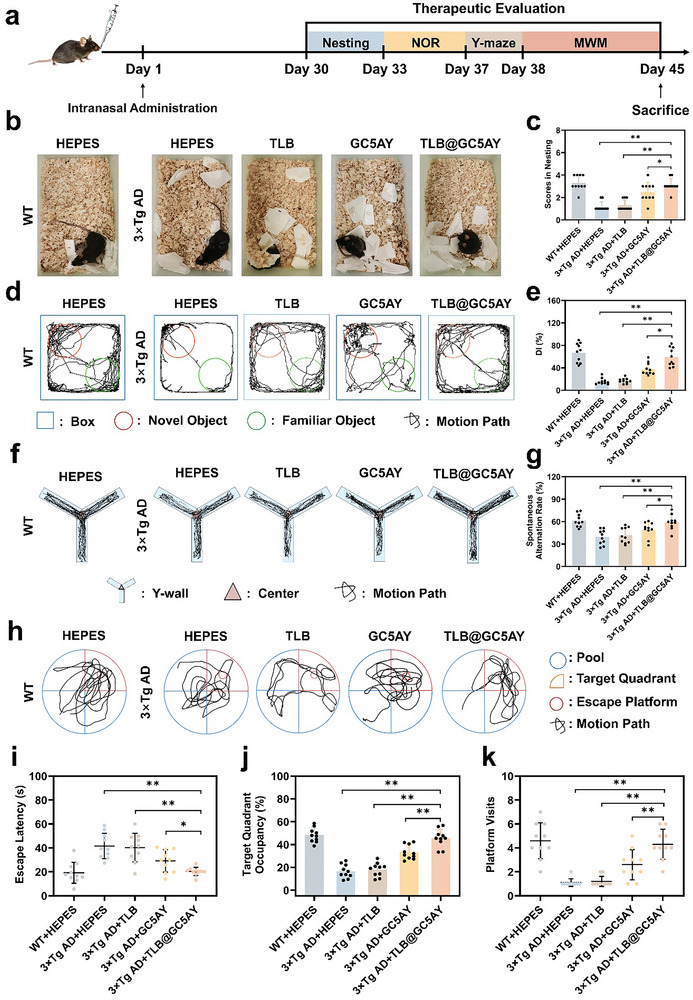
Attenuation of cognitive impairment in 3×Tg AD mice by intranasal administration of TLB@GC5AY. a) Time schedule of the experimental procedure. b) The representative of digital photographs demonstrating the nesting status of mice, and c) the corresponding nesting scores. d) Representative searching paths of mice for NOR test, and e) the corresponding calculated DI. f) Representative Y‐maze track, and g) the corresponding calculated spontaneous alternation rate. h) Representative swimming track, i) escape latency, j) target quadrant occupancy, and k) platform visits of mice on the test day in MWM test. All data were expressed as mean ± s.d. from ten mice in one group. The significance levels, with ^**^ *p* < 0.01, and ^*^ *p* < 0.05 versus 3×Tg AD + HEPES group, were analyzed by one‐way ANOVA with Tukey's test.

Based on the promising experimental results described above, we further evaluated the therapeutic effects of different formulations on improving the cognitive dysfunction in 3×Tg AD mice, including TLB, GC5AY as well as TLB@GC5AY. A previously ineffective dose of TLB was used to verify the advantages for using GC5AY as nasal drug delivery carrier.^[^ ^32^ ^]^ Following intranasal administration for 1 month, hippocampal‐dependent cognitive assessments were systematically administered through validated behavioral paradigms, including nesting test, novel object recognition test (NOR), Y‐maze test, and Morris water maze (MWM) (**Figure** **3**a).^[^ ^33^ ^]^

First, in the nesting test, the evaluation criteria for nesting scores encompass nest completion, material fragmentation, and structural integrity. Attenuation of these neurobehavioral indices correlates with deficits in affective states, cognitive function, and social interaction paradigms of the animal.^[^ ^34^ ^]^ As shown in Figure 3b,c, the 3×Tg AD mice in HEPES‐treated control group exhibited significantly lower nesting scores compared to WT group. While treatment with GC5AY and TLB@GC5AY increased the value by 2.1 and 2.6 times, respectively, indicating the substantial improvement in nesting ability and social behavior of AD mice after administration. Next, the NOR and Y‐maze tests revealed that, the mice in both GC5AY and TLB@GC5AY‐treated groups displayed increased interest in novel objects (Figure 3d,f), with discrimination index (DI) elevated by 2.4 and 3.8 times, and spontaneous alternation rate improved by 25% and 50%, respectively, compared to control (Figure 3e,g). Coincidentally, the MWM test showed that, the escape latency of the mice treated with GC5AY and TLB@GC5AY decreased by 30% and 51% (Figure 3i), while the time spent in the target quadrant increased by 2.0 and 2.8 times (Figure 3j), and the number of platform crossings increased by 2.4 and 3.9 times, respectively, compared to control (Figure 3k). These findings collectively suggested that both GC5AY and TLB@GC5AY could significantly enhance the spatial learning and memory function of AD mice.

Notably, TLB@GC5AY treatment outperformed single GC5AY in all the above behavioral tests, while TLB alone exhibited negligible therapeutic activity, with the value in this group demonstrating no significant differences compared to 3×Tg AD + HEPES group (Figure 3b–k). This was primarily due to the low nasal mucosal permeability of TLB that severely limited its brain entry, whereas GC5AY effectively overcame the nasal mucosal barrier, improving the delivery of the drug to the brain and facilitating the active compound reaches the target site in therapeutically relevant concentrations. These findings highlighted the essential role of GC5AY as a nasal delivery carrier for increasing the therapeutic efficacy of drug. As further coupled with its inherent therapeutic activity for achieving combinational therapy, the TLB@GC5AY formulation thus demonstrated significantly improved treatment outcomes of AD.

### 2.5 Reducing Aβ Plaque Content, Oxidative Stress, and Neuroinflammation in the Brain of 3×Tg AD Mice via Intranasal Administration of TLB@GC5AY

After the behavioral tests, the mice were euthanized and the mechanisms underlying the beneficial effect of these formulations were further investigated. Notably, since intranasal administration of TLB alone at the tested dosage demonstrated no significant therapeutic efficacy, this treatment group was excluded from the subsequent studies, with only GC5AY and TLB@GC5AY involved.

**Figure 4:**
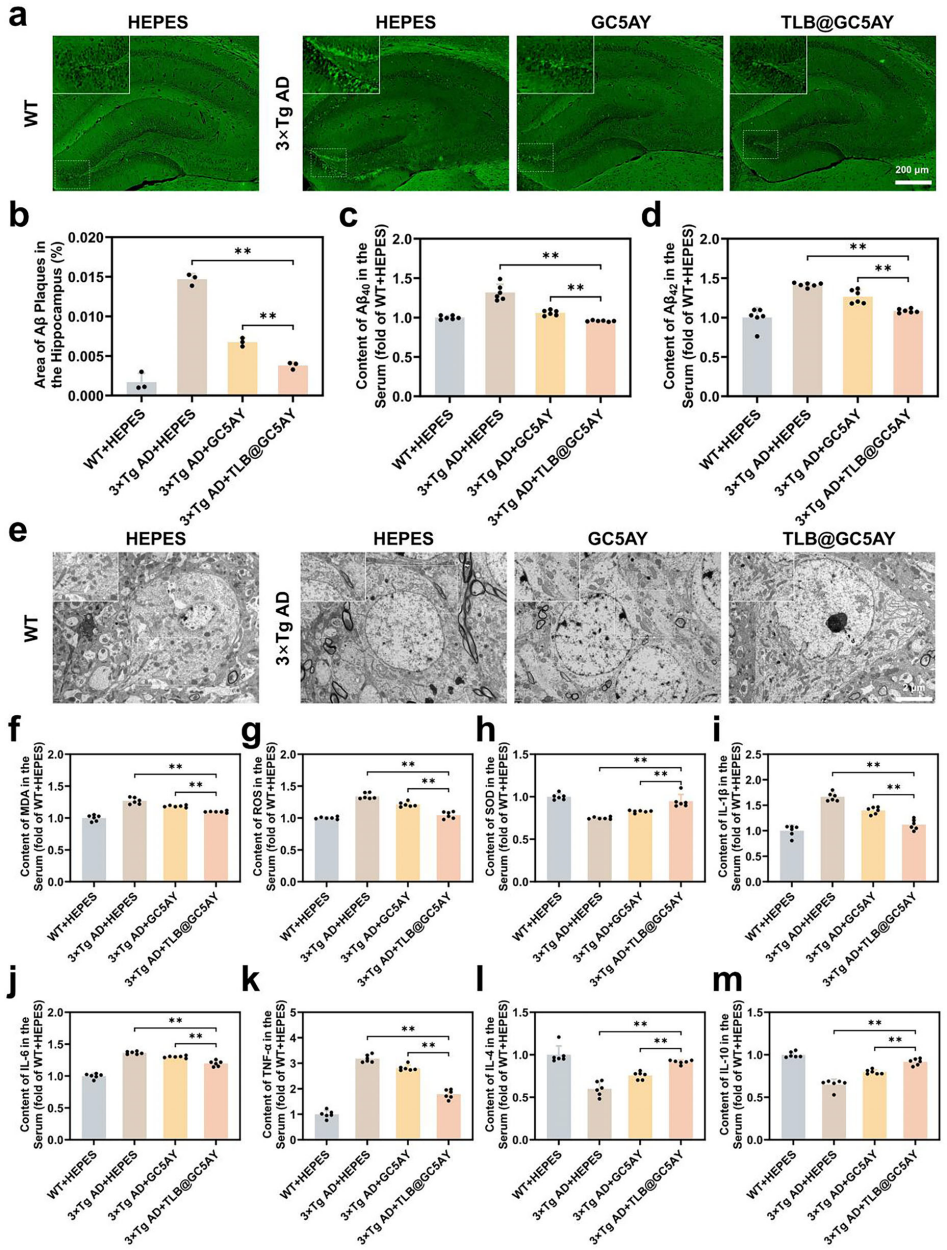
Reduction of Aβ plaque, oxidative stress, and neuroinflammation levels in the brain of 3×Tg AD mice by intranasal administration of GC5AY and TLB@GC5AY. a) The representative fluorescent images of ThioS staining in the Dentate gyrus (DG) regions of the hippocampus (scale bar = 200 µm), and b) the corresponding area fraction of ThioS (*n* = 3). c) Content of Aβ_40_, and d) Aβ_42_ in the serum (*n* = 6). e) Representative images of ultrastructural changes of hippocampal mitochondria observed by TEM (scale bar = 2 µm). Serum content of f) MDA, g) ROS, h) SOD, i) IL‐1β, j) IL‐6, k) TNF‐α, l) IL‐4, and m) IL‐10 (*n* = 6). The significance levels, with ^**^ *p* < 0.01 versus 3×Tg AD + HEPES group, were analyzed by one‐way ANOVA with Tukey's test.

The brain tissue and serum samples of the mice were collected for analyzing the major biomarkers, including levels of Aβ plaque, oxidative stress and neuroinflammation. First, Thioflavin S (ThioS) staining was used to visually assess Aβ deposition in the brain.^[^ ^35^ ^]^ Compared to the WT group, the 3×Tg AD + HEPES group exhibited a marked increase in Aβ deposition in the hippocampal region, as evidenced by the substantial fluorescent signals observed. However, after treatment with GC5AY and TLB@GC5AY, the fluorescence intensity was evidently decreased by 54% and 74%, respectively, compared to the control (**Figure** **4**a,b). And enzyme linked immunosorbent assay (ELISA) also demonstrated a consistent reduction in serum levels of Aβ_40_ and Aβ_42_ after treatment (Figure 4c,d), which collectively indicated that GC5AY and TLB@GC5AY could significantly decrease the Aβ plaque content of AD mice. Next, TEM was used to observe mitochondrial ultrastructure change to determine mitochondrial damage in the hippocampal neurons of AD mice. As shown in Figure 4e, the 3×Tg AD + HEPES group exhibited obvious mitochondrial ultrastructural damage, including swollen mitochondrial matrix, shortened and disorganized cristae, and decreased matrix electron density. While treatment with GC5AY and TLB@GC5AY significantly reversed these changes.

In addition, the levels of oxidative stress markers, antioxidant enzymes, and inflammatory cytokines in the serum were measured by ELISA. And as the results showed, compared to the 3×Tg AD + HEPES group, GC5AY and TLB@GC5AY treatment reduced the level of malondialdehyde (MDA) by 6.6% and 13%, ROS by 9.7% and 22%, pro‐inflammatory cytokines tumor necrosis factor (TNF‐α) by 12% and 44%, interleukin (IL)‐1β (IL‐1β) by 16% and 33%, and IL‐6 by 4.7% and 12%, respectively. Furthermore, the content of superoxide dismutase (SOD) was increased by 10% and 27%, anti‐inflammatory cytokines IL‐4 by 26% and 53%, and IL‐10 by 22% and 41%, respectively (Figure 4f–m). These findings collectively indicated that both GC5AY and TLB@GC5AY improved mitochondrial damage in neurons, alleviated oxidative stress, and suppressed the inflammatory response in AD mice. Notably, as consistent with the results obtained in behavioral tests, TLB@GC5AY suggested a greater degree of improvement compared to GC5AY across all measured indicators, which could be attributed to the enhanced treatment efficacy achieved through combinational therapy that effectively disrupted the mutual promotion between multiple pathologic pathways.

### 2.6 Alleviating Neuronal Apoptosis in the Hippocampus of 3×Tg AD Mice via Intranasal Administration of TLB@GC5AY

**Figure 5:**
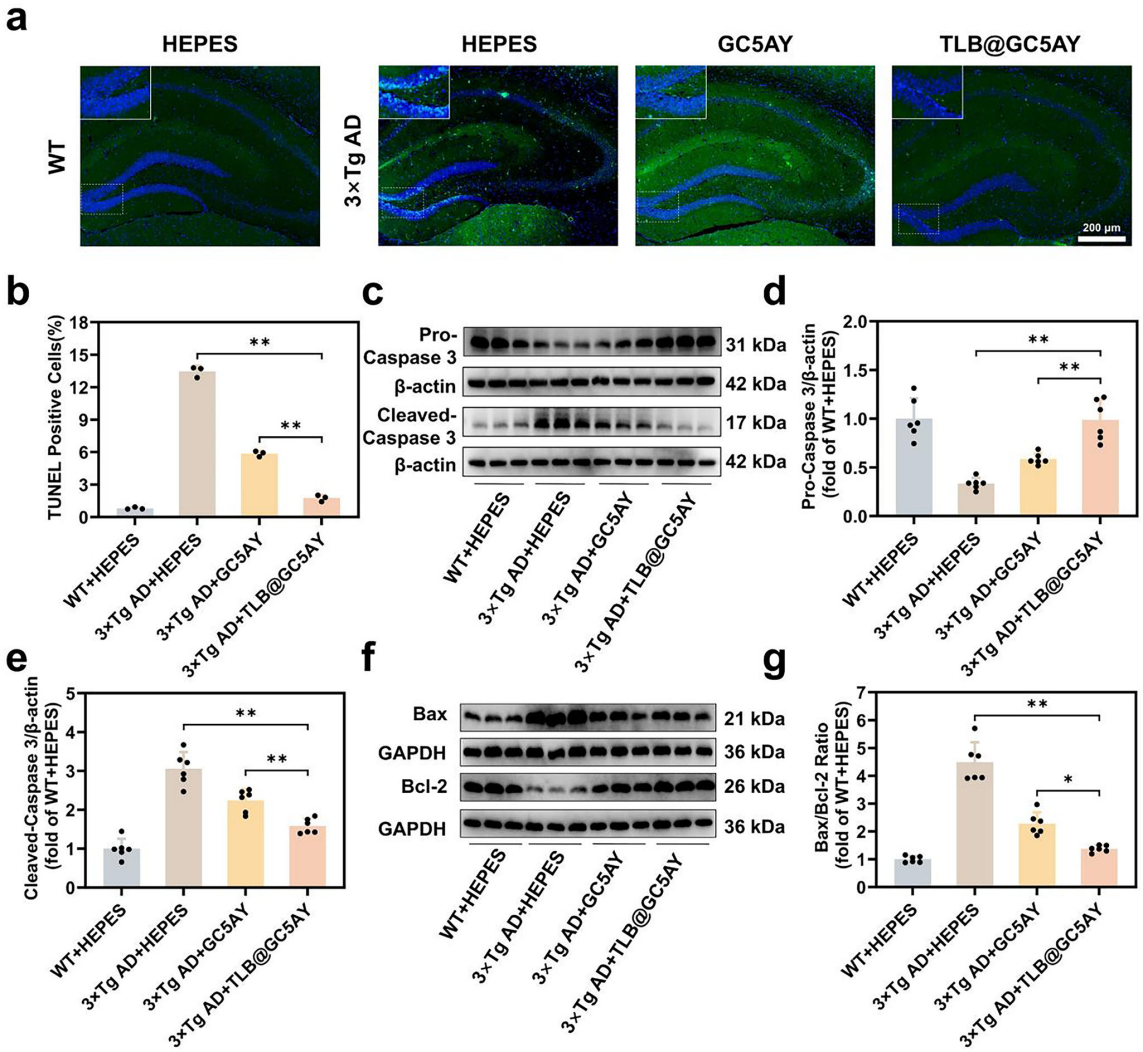
Alleviation of neuronal apoptosis in the hippocampus of 3×Tg AD mice via intranasal administration of TLB@GC5AY. a) Representative TUNEL images in the DG region of hippocampus (scale bar = 200 µm), and b) the corresponding TUNEL positive cell percentages (%) (*n* = 3). c) Representative WB bands of Pro‐Caspase 3 and Cleaved‐Caspase 3 in the hippocampus. Quantitative analysis of d) the Pro‐Caspase 3/β‐actin, and e) Cleaved‐Caspase 3/β‐actin (*n* = 6). f) Representative WB bands of Bax and Bcl‐2 in the hippocampus. g) Quantitative analysis of the Bax/Bcl‐2 ratio (*n* = 6). The significance levels, with ^**^ *p* < 0.01 and ^*^ *p* < 0.05 versus 3×Tg AD + HEPES group, were analyzed by one‐way ANOVA with Tukey's test.

Subsequently, we further investigated the effect of GC5AY and TLB@GC5AY on alleviating the neuronal apoptosis in 3×Tg AD mice, which are characteristic pathophysiological changes associated with cognitive decline in AD.^[^ ^36^ ^]^ TdT‐mediated dUTP nick‐end labeling (TUNEL) staining results showed that TUNEL‐positive cell number obviously decreased after treatment with GC5AY and TLB@GC5AY (by 56% and 87%, respectively), indicating that both of them could effectively reduce the neuronal apoptosis in 3×Tg AD mice, while TLB@GC5AY exhibited superior anti‐apoptotic effects compared to GC5AY (**Figure** **5**a,b). In addition, Aβ could facilitate cytochrome c release from impaired mitochondria, causing the cleavage and activation of Caspase 3, which eventually results in neuronal apoptosis and neuronal loss.^[^ ^37^ ^]^ Notably, the pro‐apoptotic protein Bax triggers cytochrome *c* release, and the anti‐apoptotic protein Bcl‐2 restrains Bax activation.^[^ ^38^ ^]^ Western blotting (WB) assay was further performed to determine apoptosis‐related proteins levels including Bcl‐2, Bax, Pro‐Caspase 3, and Cleaved‐Caspase 3. The results showed that, compared to the 3×Tg AD + HEPES group, the Bax/Bcl‐2 ratio in the hippocampus of mice treated with GC5AY and TLB@GC5AY was decreased by 49% and 69%, respectively. Likewise, the Cleaved Caspase‐3 protein level was decreased by 27% and 48%, while the expression of Pro‐Caspase 3 was increased by 1.7 and 2.9 times, respectively (Figure 5c–g). Consistently, TLB@GC5AY exhibited a greater improvement than GC5AY alone. These findings collectively demonstrated that the combinational therapy produced an enhanced effect, ultimately exerting beneficial effects in reducing hippocampal neuronal apoptosis in AD mice.

### 2.7 Biosafety Profiles of GC5AY and TLB@GC5AY Confirmed through In Vivo Evaluation

**Figure 6:**
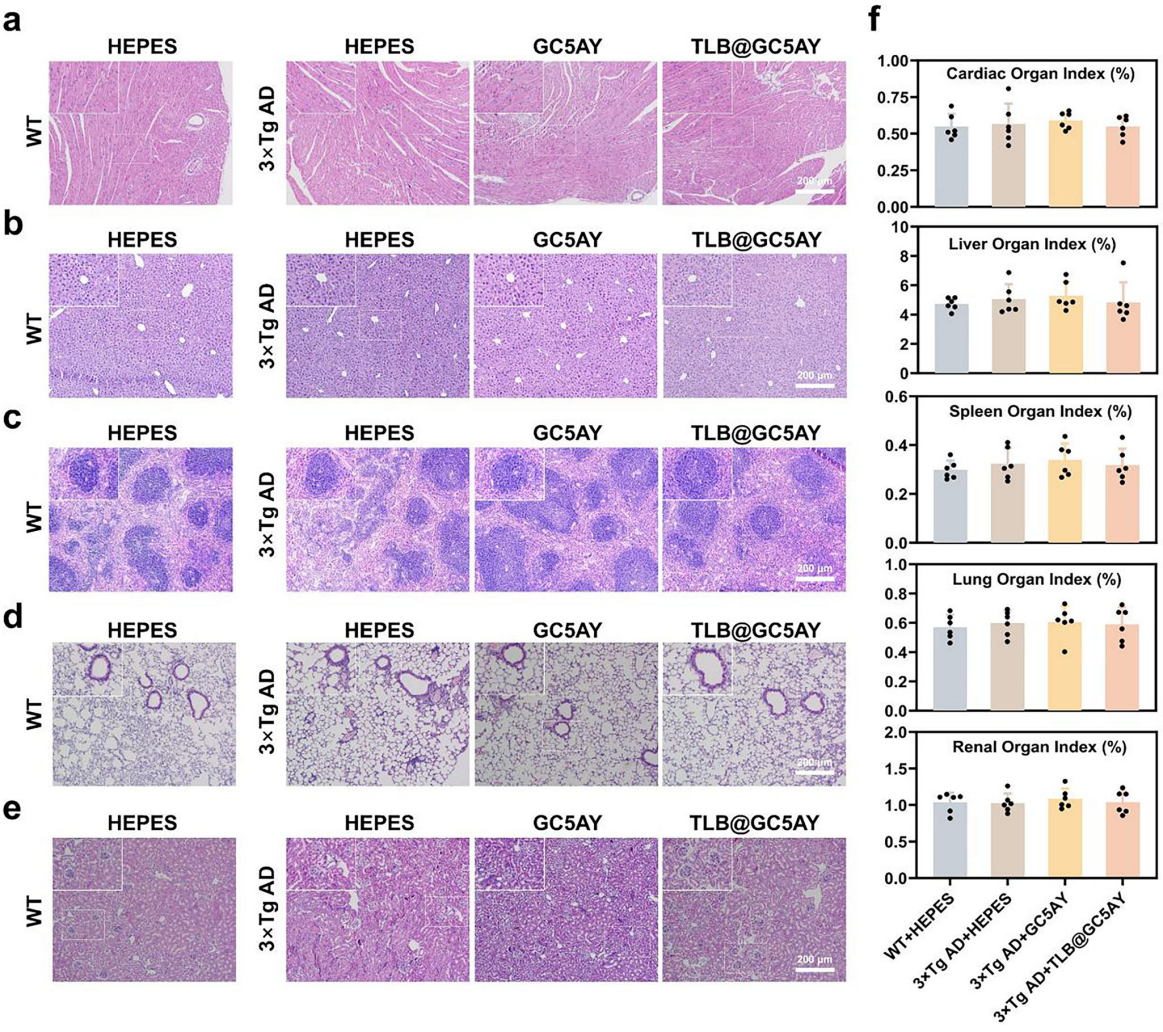
Biosafety profiles of TLB@GC5AY. Representative images of a) cardiac, b) liver, c) spleen, d) lung, and e) renal by H&E staining of GC5AY and TLB@GC5AY‐treated mice. Organ index of f) cardiac, g) liver, h) spleen, i) lung, and j) renal. All data were expressed as mean ± s.d. from samples of six mice in one group.

The above‐mentioned results have demonstrated the significant therapeutic effects of GC5AY and TLB@GC5AY in 3×Tg AD mice. And comprehensive toxicological profiling through multiparametric analysis in animal model was conducted to establish biosafety profiles, which is vital for clinical applications in the future. After treatment with GC5AY and TLB@GC5AY, the hematoxylin and eosin (H&E) staining of the heart, liver, spleen, lungs, and kidneys, as well as the organ index of each tissue, showed no pathological abnormalities, indicating that both formulations had good biological safety (**Figure** **6**a–f).

## 3 Conclusion

In conclusion, our study highlights the promising potential of GC5AY as a single‐molecule pleuripotent scaffold for combinational therapy of AD via intranasal administration. Due to its small size, positive charge, and desirable amphiphilicity, GC5AY could effectively cross the nasal mucosal barrier, and thereby serve as a nasal delivery carrier for enhancing the brain entry of drugs. Meanwhile, it also demonstrated multiple therapeutic effect on AD, including inhibiting Aβ fibrosis and scavenging ROS. Hence, by loading the neuroprotective agent TLB into the cavity of GC5AY, the formed TLB@GC5AY formulation exhibited pronounced therapeutic efficacy, which significantly improved cognitive deficits in the 3×Tg AD mouse model, leading to a marked reduction in Aβ plaque deposition, oxidative stress, neuroinflammation, and neuronal apoptosis. This integrated therapeutic approach, targeting multiple key pathological processes associated with AD, provides a more effective strategy than focusing on a single pathway. Importantly, both GC5AY and TLB@GC5AY exhibited favorable in vivo biosafety profiles, providing a solid foundation for clinical translation. Furthermore, relying on the modular characteristics of host‐guest interactions, GC5AY is expected to be developed into a platform, with its functional boundaries set to be further expanded by loading different drugs. This study has not only developed an innovative intranasal drug delivery system for the multi‐targeted treatment of AD, but also constructed a technical framework that can be extended to other central nervous system diseases requiring multi‐targeted therapy (such as Parkinson's disease and stroke), demonstrating significant clinical translation value. Future research will focus on optimizing various drug combinations to improve therapeutic efficacy, verifying their long‐term therapeutic effects in more advanced animal models, and establishing an evaluation system that is more closely aligned with clinical practice.

## 4 Experimental Section

### Materials

All reagents and solvents were commercially available and used as received unless otherwise specified purification. Aβ_42_ peptide, 1,1,1,3,3,3‐hexafluoroisopropanol (HFIP) were purchased from Macklin (Shanghai, China). TLB was purchased from Solarbio (Beijing, China). Fl was purchased from Tokyo Chemical Industry (Shanghai, China). ThT was purchased from Sigma‐Aldrich (Shanghai, China). Proclean 300 (ST853), BCA protein assay kit (P0010), total superoxide dismutase assay kit with WST‐8 (S0101S) were purchased from Beyotime (Shanghai, China). Hydroxyl radical scavenging rate assay kit (AK318) was purchased from Bioss (Beijing, China). ThioS dye was purchased from Servicebio (Wuhan, China). One‐step TUNEL apoptosis assay kit (C1008) was purchased from Beyotime (Shanghai, China). ELISA kits for Aβ_40_ (RJ16877), Aβ_42_ (RJ16878), MDA (RJ16984), ROS (RJ17213), SOD (RJ17004), IL‐1β (RJ16944), IL‐6 (RJ16958), TNF‐α (RJ17929), IL‐4 (RJ16956), and IL‐10 (RJ16932) were purchased from Shanghai Renjie Bioengineering Institute (Shanghai, China). Primary antibodies against Bax (ab32503), Bcl‐2 (ab182858), Cleaved‐Caspase 3 (ab214430), Pro‐Caspase 3 (ab32499) were purchased from Abcam (Cambridge, UK). Primary antibodies against GAPDH (60004‐1‐1g), and β‐actin (66009‐1‐1g) were purchased from Proteintech (Wuhan, China). 5,11,17,23,29‐pentaguanidinium‐31,32,33,34,35‐penta(4‐methylpentloxy)calix[5]arene (GC5AY) was synthesized and purified according to previous literature.^[^ ^19a^ ^]^

### Apparatus

Steady‐state fluorescence measurements were recorded in a conventional quartz cell (light path 10 mm) on a Cary Eclipse equipped with a Cary single‐cell Peltier accessory. The absorbance of samples in anti‐oxidation assays was measured using a microplate reader (Agilent Bio Tek Cytation5). UV–vis spectra were recorded in a quartz cell (light path 10 mm) on a Cary 100 UV–vis spectrophotometer equipped with a Cary dual‐cell peltier accessory. Dosy NMR spectra were measured using a Bruker AV400 nuclear magnetic resonance spectroscopy instrument. Dynamic light scattering was examined on a laser light scattering spectrometer (NanoBrook 173plus and Brookhaven ZetaPals) equipped with a digital correlator at 659 and 532 nm, respectively, at a scattering angle of 90. The morphology of Aβ_42_ was examined by TEM (HITACHI HT7700 Exalens). The TopScan behavioral analysis system (Version 3.00) was used to record and analyze mouse behavioral experiments. The images of fluorescence staining were captured using laser scanning confocal microscope (Leica TCS SP8, Germany). The ultrastructure of mitochondrion was carefully examined under TEM (JEM‐1400Flash, JEOL, Tokyo, Japan) to analyze the impairment of mitochondrion. The sample OD values in the ELISA experiments were measured using a full‐wavelength microplate reader (Multiskan, USA). Hitachi HT7800 TEM (120 kV accelerating voltage), Bio‐Rad ChemiDoc MP, USA for western blotting, and IVIS Spectrum CT for fluorescence imaging. The light microscope (Olympus BX43, Tokyo, Japan) was used to examine the histopathological changes of organs.

### LogP Measurement

A certain amount of GC5AY (1.0 µmol) was dissolved in 4.0 mL n‐octanol‐saturated HEPES buffer (10 mm, pH 7.4). Then, 900 µL HEPES buffer‐saturated n‐octanol was added. The mixture was shaken in an oscillator at 25 °C for 48 h. After standing, it was centrifuged at 12000 rpm for 10 min. The upper oil phase was discarded, and the concentration of GC5AY in the lower aqueous phase was determined using the UV–vis absorbance standard curve, established by adding certain amount of GC5AY in n‐octanol‐saturated HEPES buffer. The logP value was calculated as log (C(n‐octanol)/C(HEPES buffer)), where C(HEPES buffer) represented the concentration in aqueous phase at partition equilibrium, and C(n‐octanol) denoted the concentration in oil phase, which could be obtained by subtracting C(HEPES buffer) from the total dissolved amount.

### Geometry Optimization

The density functional theory calculations were conducted by Gaussian 16 program, and performed using the SMD solvation model. Geometry optimization and frequency calculations were executed at the B3LYP/6‐31G(d) level of theory incorporating Grimme's D3 dispersion correction. Geometries were optimized without any constraints, adhering to the default convergence criteria of the Gaussian 16 software. The optimized geometry exhibited absence of imaginary frequencies.

### Fluorescence Titrations

First, the *K*_a_ values between GC5AY and the reporter dye (Fl) was measured by direct fluorescent titration. Specifically, a mixed solution containing both GC5AY (100 µm) and Fl (1.0 µm) was sequentially added into 2.5 mL Fl (1.0 µm) solution in a quartz cuvette. The fluorescence intensity (*λ*_ex_ = 494 nm, *λ*_em_ = 512 nm) was recorded before the first addition and after every addition until a plateau was reached. The *K*_a_ values were fitted out from the titration curve according to the 1:1 host‐guest binding stoichiometry. Subsequently, the *K*_a_ values of GC5AY toward TLB or Aβ_42_ were measured by the competitive fluorescent titrations. Specifically, the competitor solution (TLB or Aβ_42_) was sequentially added to the solutions of reporter pairs (GC5AY and Fl) with concentration already known. Certain amount of GC5AY and Fl was also added in the competitor solution to keep the concentration of reporter pairs constant throughout the course of titrations. The *K*_a_ values between the competitor and GC5AY were obtained by fitting fluorescent intensities according to the *N*:1 competitive binding model. For TLB, the *N* was fixed as 1, which means that one GC5AY bond with one TLB through the titration. And for Aβ_42_, the *N* was fixed as 2, which means that there were two GC5AY together bond with one Aβ_42_. And since the *K*_a_ for one GC5AY binding with one Aβ_42_ was (1.3 ± 0.1) × 10^6^ M^−1^, the overall *K*_a_ between GC5AY and Aβ_42_ was obtained by squaring this value, and determined to be (1.7 ± 0.3) × 10^12^ M^−2^. All the fluorescence titrations were performed in HEPES buffer (10 mm, pH 7.4) at 25 °C.

### ThT Fluorescence Assays—Inhibition of Aβ_42_ Aggregation

First, the Aβ_42_ peptide was dissolved in HFIP at a concentration of 1.0 mg mL^−1^. After shaken at room temperature for 24 h, it was stored as a stock solution at −20 °C before use. For test, a certain volume of the Aβ_42_ solution was dried under N_2_ flow to form a thin film, which was then redissolved in 20 mm NaOH solution. Subsequently, the solution was diluted 50 times in HEPES buffer (10 mm, pH 7.4) with or without GC5AY (20 µm). The final concentration of Aβ_42_ was determined to be 20 µm using a BCA protein assay kit. The solution was then incubated through continuous orbital shaking with 200 rpm at 37 °C for 96 h. At various time points during the incubation, 60 µL of the solution was taken out and mixed with 540 µL ThT stock solution (25 µm) in HEPES buffer (10 mM, pH 7.4). The fluorescence intensity (*λ*_ex_ = 440 nm, *λ*_em_ = 480 nm) was recorded, and normalized as a percentage relative to the control group, with the initial value set at 0% and the maximum value of the control group set at 100%.

### ThT Fluorescence Assays—Disintegration of Aβ_42_ Fibrils

The pre‐prepared Aβ_42_ fibrils were obtained by incubation through continuous orbital shaking with 200 rpm at 37 °C for 96 h. Subsequently, GC5AY solution was added with a final concentration of 20 µm, and the mixed solution was further incubated with continuous shaking for 20 h. At various time points during the incubation, 60 µL solution was taken out and mixed with 540 µL ThT stock solution (25 µm) in HEPES buffer (10 mm, pH 7.4). The fluorescence intensity (*λ*_ex_ = 440 nm, *λ*_em_ = 480 nm) was then recorded, and normalized as a percentage of the control group, with the initial value in control group set to be 100% and value of pure ThT solution set to be 0%.

### Antioxidant Capacity Detection—OH Scavenging Activity

The ·OH scavenging efficiency of GC5AY was determined using a hydroxyl radical scavenging rate assay kit (Bioss, AK318), according to the procedure given in the operation manual. Specifically, ·OH was generated through the Fenton reaction (50 µm FeSO_4_ + 1 mm H_2_O_2_) of H_2_O_2_ and Fe^2+^, which would then cause the oxidation of Fe^2+^ in ferroin to Fe^3+^ and the corresponding decreased absorbance at 536 nm. The scavenging efficiency of GC5AY at different concentrations was determined by normalizing the absorbance in each with respect to the control.

### Antioxidant Capacity Detection— ⋅O_2_^−^ Scavenging Activity

The ⋅O_2_^−^ scavenging efficiency of GC5AY was determined using a total superoxide dismutase assay kit with WST‐8 (Beyotime S0101S), according to the procedure given in the operation manual. Specifically, ⋅O_2_^−^·was generated through the catalysis of xanthine by xanthine oxidase (0.1 U mL^−1^), and further reacted with WST‐8 resulting in the production of formazan dye (*λ*_abs_ = 450 nm). The scavenging efficiency of GC5AY at different concentrations was determined by normalizing the absorbance in each with respect to the control.

### In Vitro Simulation of Transmembrane Transport Using Transwell System

Calu‐3 cells (ATCC HTB‐55) were seeded at a density of 1 × 10^4^ cells well^−1^ in the upper chamber (24 well, pore size: 0.4 µm, NEST). The culture medium was changed daily, and the TEER value was measured every 2 days until it reached 1000 Ω·cm^2^. The upper chamber was added with 200 µL different solution at various groups (dye concentration of 100 µm), while the lower chamber received 800 µL fresh medium. At 1.5, 6, 12, 24, and 36 h, 100 µL of liquid was collected from the lower chamber, respectively. For the NiR@Liposome and NiR@GC5A12C groups, the collected liquid was mixed with 2.4 mL HEPES solution. For the Free Fl, Fl@GC5A12C, and Fl@GC5AY groups, the collected liquid was combined with 2.4 mL HEPES solution containing 100 mm ATP to release the fluorescent molecules from the calixarene cavity into the solution. Fluorescence was quantified using the spectrofluorometer, and membrane transport efficiency was then calculated by dividing the amount of fluorescent molecules in the lower chamber (determined using the standard curve) by the amount totally added.

### In Vivo Optical Imaging

Male C57BL/6J mice (8‐week‐old) were randomly divided into the following 5 groups: 1) AlPcS_4_, 2) AlPcS_4_@GC5AY, 3) AlPcS_4_@GC5A12C, 4) NiR@GC5A12C, and 5) NiR@Liposome (1,2‐distearoyl‐sn‐glycero‐3‐phosphorylcholine) (*n* = 5 per group). After one week of adaptive feed, the mice were intranasally administered with different formulations (10 µL per mouse) and the fluorescence intensity in the brain of each mouse was measured at 0, 2, 4, 8, 12, and 24 h after administration, using a Nightowl LB 983 in vivo imaging system (Berthold, Germany). The concentration of each formulation was 100 µm for AlPcS_4_, 100 µm/100 µm for AlPcS_4_@GC5AY and AlPcS_4_@GC5A12C. The intensity of NiR in NiR@Liposome and NiR@GC5A12C was kept same by being measured through fluorescence spectrophotometer. All these formulations were prepared in HEPES buffer (10 mm, pH 7.4). All animal experiments conducted in this study were approved by the Ethics Committee of Zunyi Medical University (approval number: ZMU22‐2303‐044).

### Behavioral Experiments

Male 3×Tg AD model mice [B6; 129‐Tg (APPSwe, tauP301L)1Lfa *Psen1^tm1Mpm^*/Mmjax] and wild‐type (WT) mice (C57BL/6J) (10‐month‐old), weighing 25−30g were randomly divided into the following five groups (*n* = 10/group): 1) WT + HEPES, 2) 3×Tg AD + HEPES, 3) 3×Tg AD + TLB, 4) 3×Tg AD + GC5AY, 5) 3×Tg AD + TLB@GC5AY. The experimental mice were all housed in specific pathogen free (SPF) animal laboratories with a quiet environment and a 12 h day/night cycle. The room temperature (22 ± 1 °C) and humidity (60 ± 2%) remained relatively constant, and the animals were able to freely drink water and food. After one week of adaptive feeding, the animals were intranasally administered with different formulations for 30 days. Specifically, the 3×Tg AD + GC5AY group and the 3×Tg AD + TLB@GC5AY group of mice received intranasal administration involved bilateral delivery of 10 µL/nostril of GC5AY or TLB@GC5AY once daily (45.8 µm), the 3×Tg AD + TLB group received intranasal administration of 10 µL of TLB once daily (45.8 µm), while the WT group and 3×Tg AD group mice received the same volume of solvent (HEPES).

### Behavioral Experiments—Nesting Test

Nesting behavior refers to the behavior of animals using materials to construct nests in specific environments, and experiments were conducted following previously methods.^[^ ^28^ ^]^ In the experiment, 10 sterile nesting paper, each measuring 1 × 5 × 5 cm, were scattered and placed in the feeding cages of the mice. Before the experiment began, the mice were allowed to adapt to the cage for 24 h. The experiment was then conducted at 7 pm to align with the circadian rhythm of mice. A double‐blind method was used to assess the nesting behavior of the mice at 3‐day post‐experiment initiation. The scoring system for the nesting behavior was as follows: 1) 0 point: no interaction with the paper or any visible bite marks, 2) 1 point: scattered pieces of paper around the cage with no distinct bite marks. 3) 2 points: paper pieces were gathered on one side of the cage, loose and without clear nesting formation, and no bite marks. 4) 3 points: paper pieces were concentrated on one side or in a corner of the cage, with a portion visibly chewed into a shallow and flat nest. 5) 4 points: most of the paper pieces were chewed and gathered into a cohesive nest, displaying a clear 3D structure and integrity.

### Behavioral Experiments—NOR Test

The NOR trial was used to assess the attention, memory, and discriminative abilities of the mice, and experiments were conducted following previously methods.^[^ ^39^ ^]^ The experiment was divided into three distinct phases with each phase separated by a 24‐h interval: 1) Habituation Phase: each mouse was placed in the experimental apparatus, which was a box measuring 50 cm in length, width, and height, devoid of any objects. The mouse was allowed to freely explore the empty apparatus for 5 min to acclimate to the environment; 2) Training Phase: two identical objects (denoted as O1 and O2) were placed in two diagonally opposite corners of the apparatus. Each mouse was introduced to the apparatus in a corner without either object and was allowed to explore freely for 5 min; 3) Recognition Phase: in this phase, one of the original objects (O1) was replaced with a novel object (N1), while the other object (O2) remained unchanged in its original position. The mouse was allowed to explore freely for 5 min, and exploratory behavior was defined as the mouse pointing its nose and/or front paw within 2 cm of any object. The percentage recognition index was calculated by the following formula:

(1) $$\mathrm{DI}=N1/\left(N1+O2\right)$$

### Behavioral Experiments—Y‐Maze Alternation Test

The Y‐maze alternation trial was used to evaluate the spatial recognition memory of mice, as described in the previous study.^[^ ^39^ ^]^ The apparatus consisted of three identical arms (30 cm **×** 10 cm **×** 20 cm), arranged in a “Y” shape. First, the mouse was placed at the start arm and allowed to freely explore the two other arms for 8 min. After returning the mouse to the same start arm, it was given 5 min to explore all three arms freely. During this phase, the number of entries into each arm was recorded. The spontaneous alternation was calculated using the formula:

(2) $$\begin{array}{c} \left[\;\left(\mathrm{number}\;\mathrm{of}\;\mathrm{actual}\;\mathrm{alternations}\right)/\left(\mathrm{number}\;\mathrm{of}\;\mathrm{total}\;\mathrm{arm}\;\mathrm{entries}2\right)\;\right]\times 100\% \end{array}$$

### Behavioral Experiments—MWM Test

The MWM trial was conducted to assess the spatial learning and memory abilities of the mice, as described in the previous study.^[^ ^39^ ^]^ The experimental apparatus consisted of a circular pool with a diameter of 120 cm and a height of 50 cm. The water temperature was maintained at 23 ± 1 °C. The pool was divided into four equal quadrants, with a platform (10 cm in diameter) submerged 1 cm beneath the water surface in one of the quadrants. First, the mice underwent a 5‐day training period where they were tasked with locating the submerged platform. The position of platform remained consistent across all trials, and the mice were allowed a maximum of 1 min to find it. If a mouse failed to find the platform within this time, it was guided to the platform by the experimenter. On the 6th day, a probe trial was performed to evaluate memory retention. The platform was removed, and the mice were allowed to swim freely in the pool for 1 min. The escape latency, time spent in the target quadrant, number of target crossings and swimming speed were determined by the analysis‐management system.

### ThioS Staining

To observe Aβ plaque in the hippocampus of mice, immunofluorescence staining was performed. First, the tissue sections were subjected to dewaxing to remove paraffin, preparing them for subsequent staining. The slides were treated with xylene, followed by rehydration through a graded alcohol series. Subsequently, 1% ThioS staining solution was added dropwise to cover the tissue sections. The slides were stained for 20 min to highlight Aβ plaque. After staining, the sections were placed into a slide soaking box containing PBS buffer and washed three times, each wash lasting 5 min, to remove excess stain. The sections were then dried gently using filter paper. After staining, neutral resin was applied to cover the sections and prevent degradation, ensuring the preservation of the immunofluorescent signal. The images were captured using laser scanning confocal microscope (Leica TCS SP8, Germany). Data analysis utilized ImageJ for ThioS quantification and SPSS 29.0 software for statistical analysis (one‐way ANOVA with Tukey's test).

### ELISA

The serum samples were collected from the mice. Following twice centrifugation at 3000 **×** g for 20 min at 4 °C, the levels of Aβ_40_, Aβ_42_, ROS, MDA, and SOD, as well as series of inflammatory cytokines (TNF‐α, IL‐1β, IL‐6, IL‐4, and IL‐10) in the serum were then measured using appropriate ELISA kits. The levels of ROS and MDA, and SOD, were used as indicators of oxidative stress.^[^ ^41^ ^]^

### TEM

The hippocampus samples were first fixed in 2.5% glutaraldehyde for 24 h. Subsequently, a secondary fixation step was performed using 1% osmium tetroxide for 2 h.^[^ ^41^ ^]^ The samples were then dehydrated through a graded series of acetone concentrations and subsequently embedded in araldite resin. Once the embedding process was complete, the samples were sectioned to a thickness of 80 nm. And the sections were counterstained with uranyl acetate and lead citrate. Finally, the ultrastructure of mitochondrion was carefully examined under a TEM (JEM‐1400Flash, JEOL, Tokyo, Japan) to analyze the impairment of mitochondrion.

### TUNEL Staining

Apoptosis of hippocampal tissue cells was assessed using TUNEL staining with a one‐step TUNEL apoptosis assay kit, following the instructions of manufacturer. Briefly, hippocampus samples were incubated with TUNEL reagent at 37 °C for 1 h. Following this, the samples were counterstained with 4′,6‐diamidino‐2‐phenylindole (DAPI) at room temperature for 8 min. Apoptotic cells were identified by the green fluorescence emitted by TUNEL‐positive nuclei, while total nuclei were stained blue with DAPI. The percentage of apoptotic nuclei was calculated by determining the proportion of TUNEL‐positive nuclei (green) relative to the total nuclei (blue). The samples were analyzed under laser scanning confocal microscope (Leica TCS SP8, Germany) for quantification and visualization of apoptosis in hippocampus.

### WB Analysis

The hippocampus samples were lysed in radio immunoprecipitation assay (RIPA) buffer supplemented with 1% protease inhibitor. The lysates were then centrifuged at 15 000 **×** g for 15 min at 4 °C. The total protein concentration in the supernatant was quantified using a BCA protein assay kit. Equal amounts of protein (30 µg) from the tissue lysates were separated on a 6−12% SDS‐PAGE gel, followed by transfer to PVDF membranes using electroblotting. The membranes were blocked with 5% (w/v) non‐fat powdered milk in TBST at room temperature for 2 h to prevent non‐specific binding. The membranes were then incubated overnight at 4 °C with primary antibodies against Bax (1:1000), Bcl‐2 (1:2000), Cleaved‐Caspase 3 (1:5000), Pro‐Caspase 3 (1:10 000), GAPDH (1:5000), and β‐actin (1:5000) at 4 °C overnight. Following primary antibody incubation, the membranes were washed and incubated with HRP‐conjugated secondary antibodies. Finally, the protein bands were visualized using an ECL Western blot detection kit (MA0186, Merck Millipore, China), and the relative intensity of the bands was quantified using the ChemiDoc MP Imaging System (Bio‐Rad Laboratories, Hercules, CA, USA). This assay allowed for the accurate assessment of protein expression levels involved in apoptosis.

### H&E Staining

Each mouse was first anesthetized before undergoing transcranial perfusion with saline, followed by perfusion with 4% paraformaldehyde (PFA) to fix the various organs (heart, liver, spleen, lungs, and kidneys). The samples were then harvested and fixed in 4% PFA overnight. Following fixation, the samples were embedded in paraffin and were sectioned into 5 µm thickness using a Leica vibrating blade slicer. Finally, the sections were then counterstained with hematoxylin and eosin to highlight the tissue morphology. The light microscope (Olympus BX43, Tokyo, Japan) was used to examine the histopathological changes of organs. Before collecting each organ, the weight of each organ needs to be recorded. The index of organ was calculated according to the following formula:

(3) $$\mathrm{Organ}\;\;\mathrm{index}\;\left(\%\right)=\mathrm{organ}\;\mathrm{wet}\;\mathrm{weight}/\mathrm{body}\;\mathrm{weight}\times 100\%$$

### Statistical Analysis

All data were presented as mean ± s.d. and were analyzed using SPSS 29.0 software. Each experiment was designed to ensure equal sample sizes, with randomization and blind analysis applied to eliminate potential bias. No data points were excluded from the statistical analysis. Statistical significance was determined using one‐way analysis of variance (ANOVA) with Tukey's test. The *p* < 0.05 was considered statistically significant.

## Conflict of Interest

The authors declare no conflict of interest.

## Supporting information



Supporting information

## Data Availability

Research data are not shared.
