# Supplementary material for: A Single‐Molecule Pleuripotent Scaffold for Combinational Therapy of Alzheimer's Disease via Intranasal Administration
Source: Adv Sci (Weinh). 2025 Aug 27;12(43):e11611. doi: 10.1002/advs.202511611 (PMC12631941; doi:10.1002/advs.202511611)
Supplement: Supplementary file 1 — Supporting information [file ADVS-12-e11611-s001.docx]

**Supporting Information**

**for**

**A Single-Molecule** **Pleuripotent Scaffold for Combinational Therapy of Alzheimer’s Disease via** **Intranasal Administration**

Jian-Mei Gao ^a,#^, Wen-Bo Li ^b,#^, Na-Na Chen ^a,c,#^, Yang Yi ^a^, Ze-Han Wang ^b^, Xian Chen ^b^, Yang-Yang Zhao ^d^, Ze-Li Yuan ^a^, Jie Gao ^a^, Yu-Chen Pan ^b^, Dong-Sheng Guo ^b,^* and Qi-Hai Gong ^a,^*

^a^ School of Pharmacy, Key Laboratory of Basic Pharmacology of Ministry of Education and Joint International Research Laboratory of Ethnomedicine of Ministry of Education, Zunyi Medical University, Zunyi 563000, China

^b^ College of Chemistry, State Key Laboratory of Elemento-Organic Chemistry, Key Laboratory of Functional Polymer Materials (Ministry of Education), Frontiers Science Center for New Organic Matter, Collaborative Innovation Center of Chemical Science and Engineering (Tianjin), Nankai University, Tianjin 300071, China

^c^ Key Laboratory of Macrocyclic and Supramolecular Chemistry of Guizhou Province, Guizhou University, Guiyang 550025, China

^d^ National Engineering Research Center of Pesticide, Nankai University, Tianjin 300071, China

* Corresponding Author.

E-mail addresses: dshguo@nankai.edu.cn (D.-S. Guo), gqh@zmu.edu.cn (Q.-H. Gong)

^#^ These authors contributed equally to this work.

**Supporting Results**

**
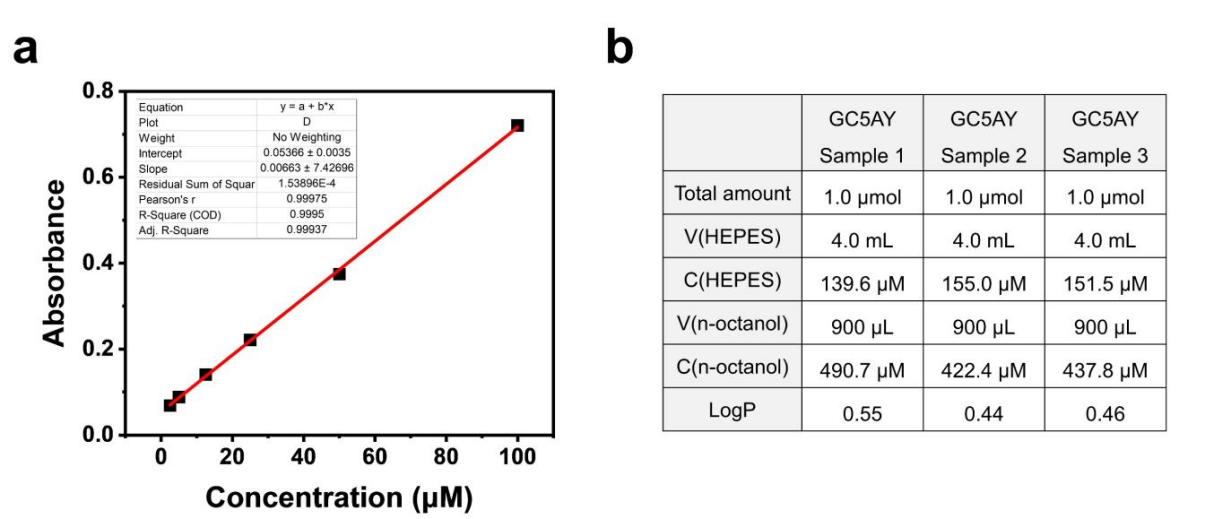
**

**Figure S1.** a) The UV-Vis absorbance standard curve of GC5AY in n-octanol-saturated HEPES buffer (10 mM, pH 7.4). b) The dissolved concentration of GC5AY in aqueous and oil phase at partition equilibrium for calculation of LogP value (*n* = 3).


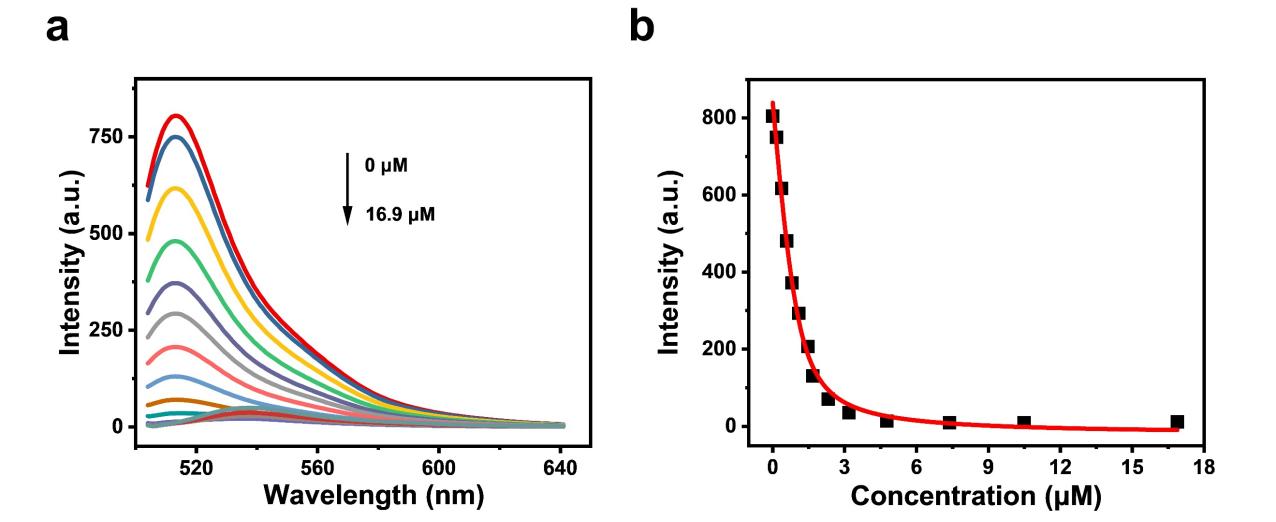


**Figure S2.** a) Direct fluorescence titration of Fl (1.0 μM) with GC5AY in HEPES buffer (10 mM, pH 7.4) at 25 °C (*λ*_ex_ = 494 nm). b) The associated titration curve at *λ*_em_ = 512 nm, fitted according to the 1:1 binding stoichiometry. The *K*_a_ was determined to be (4.4 ± 0.6) × 10^6^ M^−1^, which was consistent with our previous report as 3.2 × 10^6^ M^−1^ measured by direct UV-Vis titration.^[1]^

**
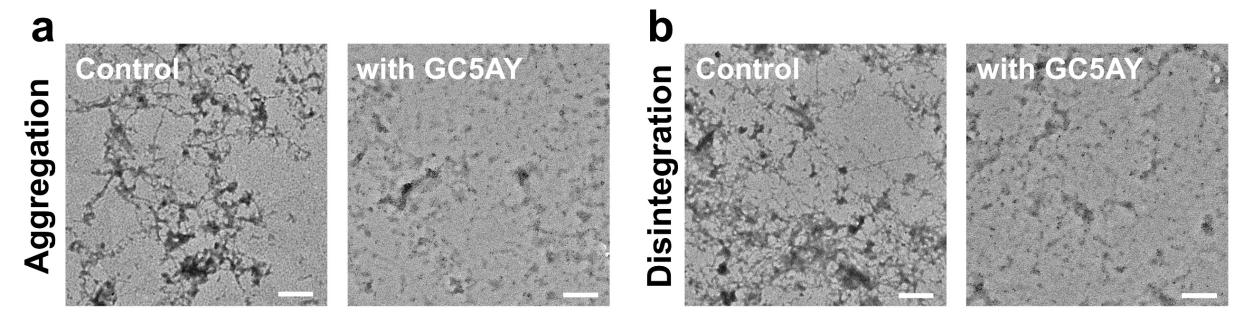
**

**Figure S3.** TEM images of a) Aβ_42_ monomer and b) pre-formed Aβ_42_ fibrils co-incubated with or without GC5AY with equal equivalent (scale bar = 300 nm).


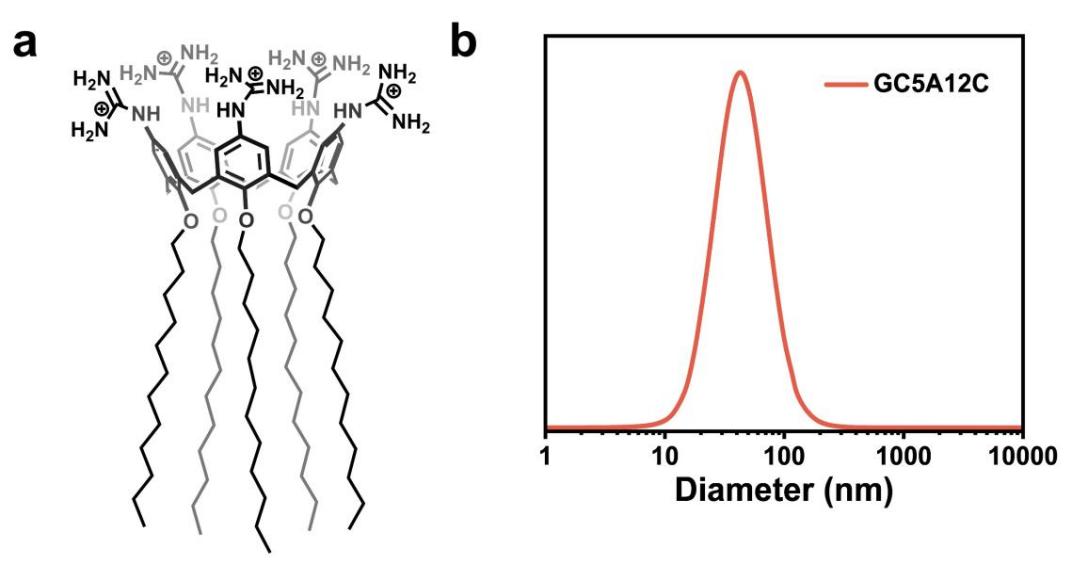


**Figure S4.** a) The chemical structure of GC5A12C. b) Diameter of GC5A12C assembly, measured by DLS, the data was obtained through number-based lognormal distribution method.

**Table S1.** The diffusion coefficients (*D*) and corresponding molecular diameter of GC5AY.

| GC5AY concentration | *D* (m^2^/s) | Diameter (nm) |
| --- | --- | --- |
| 100 μM | 1.76 × 10^−10^ | 1.39 |
| 1.0 mM | 7.75 × 10^−12^ | 31.5 |

**Note:** The CAC value of GC5AY was reported to be 400 μM.^[2]^ So GC5AY at a concentration of 100 μM was at a single-molecule state, and the diameter was for one molecule. While GC5AY at a concentration of 1.0 mM formed an assembly, which demonstrated a larger diameter.

**Reference**

[1] Z. Zheng, W.-C. Geng, J. Gao, Y.-Y. Wang, H. Sun, D.-S. Guo, *Chem. Sci.* **2018**, 9, 2087.

[2] J. Gao, J. Li, W.-C. Geng, F.-Y. Chen, X. Duan, Z. Zheng, D. Ding, D.-S. Guo, *J. Am. Chem. Soc.* **2018**, 140, 4945.
